# Evolution of a Systematic Approach to Smoking Cessation in Ontario’s Regional Cancer Centres

**DOI:** 10.3390/curroncol29070365

**Published:** 2022-06-30

**Authors:** Erin Cameron, Vicki Lee, Sargam Rana, Mohammad Haque, Naomi Schwartz, Sahara Khan, Rebecca Truscott, Linda Rabeneck

**Affiliations:** 1 Prevention & Cancer Control, Ontario Health (Cancer Care Ontario), Toronto, ON M5G 2L3, Canada; vicki.lee@ontariohealth.ca (V.L.); sargam.rana@ontariohealth.ca (S.R.); mohammad.haque@ontariohealth.ca (M.H.); naomi.schwartz@ontariohealth.ca (N.S.); sahara.khan@ontariohealth.ca (S.K.); rebecca.truscott@ontariohealth.ca (R.T.); linda.rabeneck@utoronto.ca (L.R.); 2 Dalla Lana School of Public Health, University of Toronto, Toronto, ON M5T 3M7, Canada; 3 Department of Medicine, University of Toronto, Toronto, ON M5S 3H2, Canada

**Keywords:** smoking cessation, program implementation, performance management

## Abstract

Smoking cessation after a cancer diagnosis can significantly improve a person’s prognosis, treatment efficacy and safety, and quality of life. In 2012, Cancer Care Ontario (now part of Ontario Health) introduced a Framework for Smoking Cessation, to be implemented for new ambulatory cancer patients at the province’s 14 Regional Cancer Centres (RCCs). Over time, the program has evolved to become more efficient, use data for robust performance management, and broaden its focus to include new patient populations and additional data collection. In 2017, the framework was revised from a 5As to a 3As brief intervention model, along with an opt-out approach to referrals. The revised model was based on emerging evidence, feedback from stakeholders, and an interim program evaluation. Results showed an initial increase in referrals to cessation services. Two indicators (tobacco use screening and acceptance of a referral) are routinely monitored as part of Ontario Health’s system-wide performance management approach, which has been identified as a key driver of change among RCCs. Due to the COVID-19 pandemic, many RCCs reported a decrease in these indicators. RCCs that were able to maintain a high level of smoking cessation activities during the pandemic offer valuable lessons, including the opportunity to swiftly leverage virtual care. Future directions for the program include capturing data on cessation outcomes and expanding the intervention to new populations. A focus on system recovery from COVID-19 will be paramount. Smoking cessation must remain a core element of high-quality cancer care, so that patients achieve the best possible health benefits from their treatments.

## 1. Introduction

The benefits of smoking cessation for people with cancer include significantly reduced mortality; evidence suggests that the risk of dying could be lowered by 30–40% by quitting smoking at the time of a cancer diagnosis [1]. There is clear evidence that continued tobacco use in people with cancer can lead to decreased treatment efficacy and safety, decreased quality of life, increased treatment-related toxicity, and an increased risk of recurrence and second primary tumors [2]. However, quitting smoking may reduce these adverse effects of tobacco use on cancer treatment and clinical outcomes. In response to this evidence, Cancer Care Ontario (now part of Ontario Health) implemented a smoking cessation program across the province’s cancer system. Over time, the program has evolved to become more efficient, use data for robust performance management, and broaden its focus to include new patient populations and additional data collection. The purpose of this paper is to describe the evolution of the program over the past decade, particularly the transition to a 3As (Ask, Advise, and Act) model and the subsequent increase in performance of quality indicators, the impact of the COVID-19 pandemic, and future directions.

### Framework for Smoking Cessation

In 2012, Cancer Care Ontario (now part of Ontario Health) introduced a Framework for Smoking Cessation, to be implemented for all new ambulatory cancer patients at the province’s 14 Regional Cancer Centres (RCCs). The design of the framework, initial implementation, and lessons learned were previously published [3]. Regional smoking cessation champions (champions) were designated at each RCC across the province to lead the implementation of the framework in their region and act as the liaison between the cancer center and Ontario Health. These local champions, along with endorsement from senior leadership, support from a provincial secretariat, and ongoing guidance from an advisory committee of smoking cessation experts, were considered key enablers of early success. RCCs received a limited amount of annual funding to support implementation of smoking cessation activities, and to ensure that smoking cessation is integrated as a key component of quality cancer care.

The framework was designed to be flexible and adapt to individual regional circumstances, which varied widely in terms of geographic area, population size, and demographics. RCCs were encouraged to adopt the internationally recognized 5As (Ask, Advise, Assess, Assist, and Arrange) model of smoking cessation [4]; however, a specific approach was not prescribed. The framework consisted of three main components: (1) standard set of program elements (e.g., target population of new ambulatory cancer patients, standardized screening question, and regular data submissions); (2) region-specific options (e.g., smoking cessation referral type and local partnerships); (3) centralized administrative support (e.g., central database with reporting and analytics). RCCs were required to maintain electronic processes to capture smoking cessation data elements, and to follow standardized data submission protocols.

In 2017, the framework was revised from the 5As to a 3As (Ask, Advise, and Act) brief intervention model, along with an opt-out approach to referrals. The revised model was based on emerging evidence, feedback from stakeholders, and an interim program evaluation. It was recognized that many healthcare providers did not intervene with smokers as often as they should, most commonly because the smoking cessation intervention was perceived as time-consuming. It may be unrealistic to expect oncology healthcare providers to deliver comprehensive tobacco dependence treatment to patients, given the time constraints in a busy clinic environment [5]. Nevertheless, there was a strong acknowledgement that smoking cessation must become a higher priority at cancer centers.

The 3As approach simplified the intervention for healthcare providers, making it briefer but no less effective. The streamlined approach was outlined for the RCCs as follows: (Ask) all new ambulatory cancer patients are screened for tobacco use in the past 6 months; (Advise) all current or recent smokers are provided personalized, empathetic advice on the benefits of quitting smoking for their cancer treatment and clinical outcomes; (Act) all current or recent smokers are offered a direct referral to a smoking cessation service for support in making a quit attempt or remaining smoke-free. The smoking cessation service could be either internal to the hospital, such as a quit coach (e.g., nurse, social worker, or pharmacist), or external in the community (such as the provincial quit line or a local program).

With the opt-out approach to referrals, healthcare providers were instructed to offer all current or recent smokers a referral to a cessation support program. This approach removes the need to assess and discuss the patient’s readiness or interest in quitting smoking, which makes the intervention less time-consuming to implement [6]. Since a minority of tobacco users will state that they are “ready to quit”, assessing readiness to quit can drastically limit access to evidence-based care when patients must opt in to receive tobacco treatment [7]. Changing the default approach to opt-out helps ensure that smoking cessation becomes a standard part of quality cancer care. As with any medical care, the patient may decline the referral if they choose; however, the default should be that they receive a referral once consent for the referral has been provided. In cancer care settings, an opt-out tobacco treatment approach may be particularly applicable due to the urgency to quit smoking before starting cancer treatments. Following the implementation of the new approach in RCCs in 2018, results showed a promising increase in the proportion of patients accepting a referral to smoking cessation services.

While the framework provides a standard to follow, each RCC implements it in their own unique way. This has led to several natural experiments and an attempt to identify best implementation practices for smoking cessation in cancer care settings [8]. For example, in 2017, the London Regional Cancer Program launched a pilot to provide free nicotine replacement therapy (NRT) to improve the low referral rates identified in an evaluation of their smoking cessation program [9,10]. The pilot demonstrated that provision of free NRT significantly improved referral rates, and most referred patients either reduced their cigarette smoking or quit completely. Another example is the Northeast Cancer Centre, which offers an intensive clinical tobacco intervention involving multiple follow-up appointments and pharmacotherapy. A study of head and neck cancer patients found that, although these patients reported high levels of nicotine dependence, many were able to successfully quit [11].

## 2. Performance Management

Using a set of standardized quality indicators, Ontario Health monitors the performance of the smoking cessation program at the provincial level and at the 14 individual RCCs, against annual performance targets. Ontario Health manages the submission, storage, analysis, and reporting of data provided by RCCs and hospitals into a provincial database. Many cancer system indicators (e.g., radiation and systemic therapy, and outpatient oncology clinic visits) are reported from this database, including smoking cessation indicators. This system-wide performance management approach has been identified as a key driver of change among RCCs.

Since 2015, Ontario Health has consistently reported on a set of smoking cessation performance metrics: (1) proportion of new ambulatory cancer patients screened for their smoking status (tobacco use screening); (2) proportion of individuals screened who were current or recent smokers; (3) proportion of current or recent smokers who were advised about the benefits of quitting smoking; (4) proportion of current or recent smokers who were recommended a referral to smoking cessation services; (5) proportion of current or recent smokers who accepted a referral to smoking cessation services (acceptance of a referral). The type of referral accepted (internal, external, or both) is also reported. More recently, in 2020, Ontario Health began collecting additional data on the reasons why patients declined a referral, although it is too early to draw any broad conclusions. Ontario Health works closely with the regional champions to address data submission and data quality issues. Ontario Health develops and leads discussions on quarterly performance with the RCCs, which allows their leadership teams to gauge their relative performance and helps identify potential gaps in their smoking cessation activities or data reporting strategies [12]. RCC-specific data quality issues are addressed collaboratively between the RCC and Ontario Health, while common concerns are brought to the attention of all champions and/or the smoking cessation advisory committee for resolution.

With over 5 years of data available, trends in the performance of smoking cessation programs in Ontario’s RCCs have emerged. Of the set of five performance metrics, two are monitored more closely as part of Ontario Health’s quarterly performance reviews within the regions: tobacco use screening and acceptance of a referral. Tobacco use screening is defined by patients who are asked, “Have you used any form of tobacco in the last 6 months?” Acceptance of a referral is defined by patients who agree that a provider can refer them to a cessation service. Our program is unable to collect information regarding whether the referral is completed or if the patient engages with the service.

The provincial performance of both tobacco use screening and acceptance of a referral increased between fiscal years 2015–2016 and 2019–2020 (Table 1). This resulted in a subsequent increase in the annual performance target for these indicators over time. In 2015–2016, the annual performance target for tobacco use screening was 70%; this was subsequently increased to 75%, and then to 80% in 2019–2020. Similarly, the annual performance target for acceptance of a referral was 20% in 2017–2018 and increased to 30% in 2019–2020. While the provincial-level performance of programs increased over time, there remained significant variability between the regions. In 2019–2020, for example, performance on the tobacco use screening indicator ranged from 31% to 99% between the lowest- and highest-performing RCCs, and the acceptance of a referral indicator ranged from 8% to 82% among the RCCs (data not shown). Reasons for low performance include staff turnover and capacity, documentation issues, changes to electronic medical record systems, and large patient volumes. Other RCCs have been able to embed and sustain a strong process for smoking cessation, such as integrating screening for smoking status into the new patient registration process and using automatic referrals to an internal cessation service.

### Impact of the COVID-19 Pandemic

**Figure 1:**
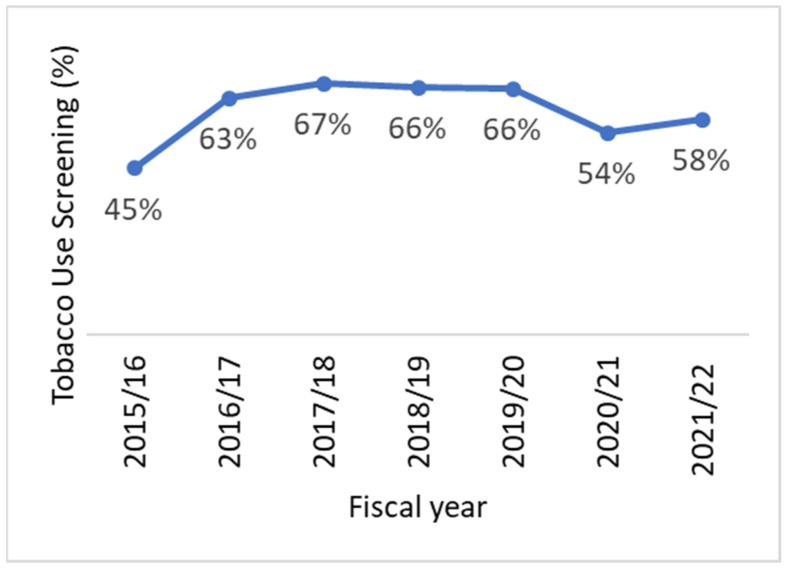
Tobacco use screening rates among new ambulatory cancer patients in Ontario RCCs, by fiscal year, 2015–2016 to 2021–2022.

**Figure 2:**
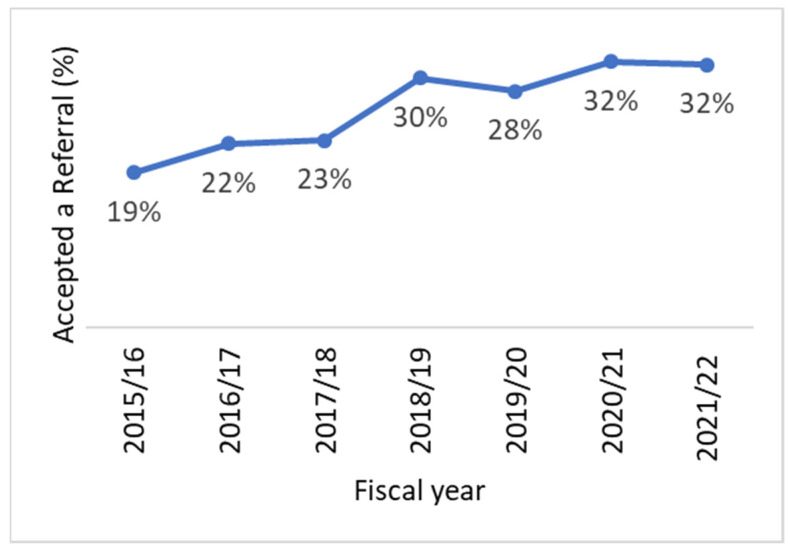
Acceptance of a referral rates among current and recent smokers in Ontario RCCs, by fiscal year, 2015–2016 to 2021–2022.

Not surprisingly, there have been significant impacts on smoking cessation activities in the RCCs due to the COVID-19 pandemic. From 2019–2020 to 2021–2022, the provincial rate for the tobacco use screening indicator declined from 66% to 58% (Figure 1 and Figure 2). However, the COVID-19 pandemic did not appear to affect the RCCs universally. While some centers saw substantially fewer patients screened for tobacco use after March 2020, others reported little to no change in their screening rates. In addition, the provincial rate for the acceptance of a referral indicator increased slightly from 2019–2020 to 2021–2022, from 28% to 32%. This is likely a reflection that the number of patients who were identified as smokers was lower, rather than a result of any policy or program changes that would have led to an increase in referrals.

Through operational discussions with the champions, several reasons for how the pandemic caused decreases in smoking cessation performance were identified. One common reason was that staff who typically support smoking cessation programs were redeployed to support frontline pandemic efforts. Another reason was disruptions to regular clinical flow that hindered the implementation of the 3As model. For example, in a center that relied on electronic tablets to screen patients for tobacco use, the ability to do so was severely impacted during the pandemic due to fewer patients attending in-person visits, as well as stopping the use of shared tablets as part of infection prevention protocols. In other centers, asking patients about smoking status and advising on smoking cessation were typically conducted during in-person visits by dedicated healthcare providers prior to the initial consult with the physician. However, with many physician visits being conducted virtually during the pandemic, the opportunity to discuss smoking cessation with patients became more difficult.

It was noted that some RCCs were able to swiftly leverage the virtual care opportunities presented by the pandemic. RCCs that already used virtual processes for their smoking cessation programs (e.g., screening for tobacco use over the phone as part of new patient registration) were able to maintain high performance rates. Other innovations occurred in response to the rapid transition to virtual care. For example, the Princess Margaret Cancer Centre implemented a “digital education prescription”—a secure online tool that allows healthcare providers to email educational resources (including pamphlets, videos, and e-learning modules) to patients and their families—to improve the delivery of smoking cessation education during COVID-19 [13].

Ontario Health is seeking to better understand the reasons why some RCCs were able to maintain their performance over the pandemic, while others were more vulnerable to its impacts, through ongoing dialogue with the champions. Recovery planning is underway to determine how best to optimize tobacco use screening rates, improve education of healthcare providers and patients about the benefits of smoking cessation, and increase the rate of referrals to cessation services. Ultimately, processes and characteristics that lead to more resilient programs, and that can withstand massive system-wide disruptions may be desirable to replicate the results more consistently across all RCCs.

## 3. Future Directions

Future directions for the program include expanding the intervention to new populations and capturing data on cessation outcomes, while keeping in mind that a focus on system recovery from COVID-19 is paramount.

### 3.1. Program Expansion

The Ontario Cancer Plan 5 (2019–2023) supports continued work in tobacco cessation under its goal to “provide effective cancer care based on best evidence,” with a key strategic objective to “expand tobacco cessation programs” [14]. In 2019, Ontario Health began work to expand the smoking cessation program to additional patient populations beyond new ambulatory cancer patients in the RCCs. The 14 regions have networks of hospitals and other agencies involved in providing cancer prevention, screening, diagnosis, treatment, and support services. Each region has identified priority populations to scale up implementation of the Framework for Smoking Cessation. Many regions will be providing smoking cessation support to people with cancer at partner hospitals, patients in the diagnostic phase, and oncology inpatients.

Over the past few years, Ontario Health has been engaging with the regions with respect to their program expansion plans. A strong focus has been on developing and managing data reporting requirements to support the same performance indicators used for ambulatory cancer patients. Many regions have expanded their programs to their identified target populations and began collecting and submitting smoking cessation expansion data in November 2021. A trial data collection period will help Ontario Health conduct initial analyses, resolve issues in data quality, and refine reporting requirements.

Many of the regions have made impressive gains in expanding their smoking cessation programs over the past couple of years. For regions that have not yet been able to expand their smoking cessation programs, Ontario Health will continue working with them to do so, while recognizing the pressures the healthcare system is facing due to the COVID-19 pandemic. Provincial expansion efforts will additionally focus on refining data reporting for the expansion populations, sharing data back with the regions in a meaningful way (e.g., standardizing reporting where appropriate), and sharing lessons learned from the different priority populations, with the goal of ensuring that every cancer patient is screened for tobacco use, and that all smokers are offered support for smoking cessation.

### 3.2. Capturing Outcome Data

Despite a robust performance monitoring process and a well-developed set of quality indicators, Ontario Health does not currently have the ability to measure smoking cessation outcomes (e.g., quit rates or reductions in smoking) for patients participating in a smoking cessation program. With funding from the Canadian Partnership against Cancer, Ontario Health is working to address this gap. A pilot project, Implementing Measures for Patient-Reported Outcomes to Verify the Effectiveness of Smoking Cessation (IMPROVE-SC), has been implemented in two RCCs to assess the feasibility of collecting smoking cessation outcome data. Leveraging an existing province-wide technology called the Interactive Symptom Assessment and Collection (ISAAC) system, Ontario Health has deployed a new tobacco use survey. As part of routine care, cancer patients at all RCCs are directed to ISAAC kiosks to complete surveys in an effort to capture patient-reported outcome and experience measures, such as reporting on cancer-related symptoms. In early 2021, the tobacco use survey was implemented on ISAAC at the two IMPROVE-SC pilot sites. This new survey asks patients approximately every 3 months about their current smoking status, the number of cigarettes smoked per day, and what methods they have used to try quitting or reducing smoking. Over time, as patients complete the tobacco use survey during the course of their cancer treatments, results have the potential to inform what proportion of patients were able to quit or reduce their smoking, and which methods were commonly used. Consequently, the survey will allow Ontario Health to evaluate the effectiveness of the smoking cessation program.

The implementation of the tobacco use survey is in the early stages, and efforts to evaluate the feasibility and acceptability of the survey are underway. The evaluation will include gathering information from patients about their experience completing the survey, as well as feedback from healthcare providers and other staff about any impact on existing clinic processes. The lessons learned from the evaluation will help inform a potential roll-out of the tobacco use survey to additional RCCs across the province. Ultimately, the data captured have the potential to be linked to other cancer system databases to assess the impact of smoking cessation efforts on health outcomes such as cancer survival, reduced side-effects of cancer treatments, and/or reduced cancer recurrence.

## 4. Conclusions

Over time, Ontario Health’s systematic approach to smoking cessation for cancer patients has evolved and responded to emerging evidence, ongoing performance monitoring and program evaluation activities, and most recently the COVID-19 pandemic. The adoption of the 3As model with an opt-out approach simplified the intervention for busy healthcare providers and resulted in increased performance on quality indicators such as tobacco use screening and acceptance of a referral rates. Ontario Health’s system-wide performance management approach has been identified as another key driver of improvements in quality indicators. Despite the overwhelming challenges due to the COVID-19 pandemic faced by the healthcare system, some RCCs have demonstrated high resilience in their continued ability to deliver smoking cessation programs as a part of quality cancer care, and even expanded their programs to identified priority populations. Future directions include assessing and sharing lessons learned from the pandemic back with all RCCs, the continued expansion of the smoking cessation program to additional priority populations in the regions, and capturing new smoking cessation outcome data that will allow for an evaluation of the program’s effectiveness. As healthcare systems are focused on recovering from the COVID-19 pandemic, smoking cessation must remain a core element of high-quality cancer care, so that patients achieve the best possible health benefits from their treatments.

## Figures and Tables

**Table 1:** Tobacco use screening and acceptance of a referral numbers and rates in Ontario RCCs, by fiscal year, 2015–2016 to 2021–2022.

	Tobacco Use Screening			Acceptance of a Referral		
Fiscal Year	Total New Patients	Number Screened (*n*)	Rate (%)	Total Patients Who Use Tobacco	Number Accepted (*n*)	Rate (%)
2015–2016	60,287	26,849	45%	4600	854	19%
2016–2017	65,313	41,435	63%	7288	1612	22%
2017–2018	66,290	44,700	67%	7588	1707	23%
2018–2019	67,231	44,593	66%	7214	2167	30%
2019–2020	67,823	44,726	66%	6855	1946	28%
2020–2021	61,405	33,299	54%	5283	1688	32%
2021–2022	65,390	37,722	58%	5930	1873	32%

## Data Availability

Ontario Health is prohibited from making the data used in this research publicly accessible if they include potentially identifiable personal health information and/or personal information as defined in Ontario law, specifically the Personal Health Information Protection Act (PHIPA) and the Freedom of Information and Protection of Privacy Act (FIPPA). Upon request, data deidentified to a level suitable for public release may be provided.
